# Opportunities for Improving Outcomes in Severe Malaria: The Role of Blood Glucose Monitoring

**DOI:** 10.4269/ajtmh.23-0178

**Published:** 2023-04-17

**Authors:** David Torres-Fernandez, Rosauro Varo, Quique Bassat

**Affiliations:** ^1^ ISGlobal, Hospital Clínic - Universitat de Barcelona, Barcelona, Spain;; ^2^ Centro de Investigação em Saúde de Manhiça (CISM), Maputo, Mozambique;; ^3^ ICREA, Pg. Lluís Companys 23, Barcelona, Spain;; ^4^ Pediatrics Department, Hospital Sant Joan de Déu, Universitat de Barcelona, Esplugues, Barcelona, Spain;; ^5^ Consorcio de Investigación Biomédica en Red de Epidemiología y Salud Pública (CIBERESP), Madrid, Spain

Severe malaria is responsible for an estimated 2–4 million cases and 619,000 deaths annually, the majority in children in Sub-Saharan Africa.^1^ Severe malaria encompasses a variety of clinical syndromes and laboratory abnormalities which differ in their frequency, prognosis, and age distribution.^2^ Hypoglycemia is one of the more underrecognized abnormalities, but it is closely associated with morbidity and mortality from malaria, and also other infections, many of which are common in low and middle-income countries.^3^ The measured case fatality rate for hypoglycemia as a complication of malaria has ranged between 3.3 and 61.5%^4^; it can be associated with seizures or coma, or occur with less overt symptomatology. Importantly, hypoglycemia is a defining condition of severe malaria, but many other severe malaria complications may increase the risk of developing hypoglycemia, particularly those associated with impaired consciousness, such as cerebral malaria (CM).^4^ Thus, the prompt evaluation of blood glucose upon first encounter with a sick malaria patient is an obligation, and its rapid correction and subsequent monitoring can be a life-saving intervention.

In their severe malaria guidelines, the World Health Organization (WHO) recommends capillary glucose measurements upon admission and every 4 hours as the minimum level of care, without further specification about monitoring duration.^5^ However, some studies suggest that hypoglycemia may be underestimated in severe malaria, including CM. Continuous glucose monitoring has shown a higher level of detection of hypoglycemia episodes during hospitalization in children with malaria (15.4%) compared to that with intermittent capillary determination (7.7%).^6^ The clinical impact of those otherwise missed episodes of hypoglycemia in terms of neurological sequelae and mortality remains to be determined. Therefore, further information is required to better understand blood glucose dynamics and develop more specific management guidelines for severe malaria.

In this issue of the *The American Journal of Tropical Medicine & Hygiene*, Chastang et al.^7^ present a retrospective study of a cohort of Malawian children with CM. They evaluated the strength of association between low glucose levels at admission/post-admission and adverse outcomes in children with CM, and also the utility of repeated glucose determinations. Their study included 1667 children aged 6 months to 14 years admitted with CM at Queen Elizabeth Central Hospital in Blantyre between September 2000 and June 2018. Patients with severe malnutrition, a typical risk factor for hypoglycemia, were excluded, and results were adjusted for changes in first-line antimalarial therapy from quinine to artemisinin-based combination therapies. Glucose levels were measured using finger-prick capillary tests on admission and every six hours for the first 48 hours. Patients were managed according to WHO and Malawian malaria treatment guidelines. Outcomes were evaluated and compared between deceased patients and survivors, and among survivors, between those who returned to neurological baseline and those who developed any neurological sequelae.

The authors found that the overall mortality rate for children with CM was 16.3%. Among survivors, 10.9% had neurological abnormalities at discharge. At least one hypoglycemic episode was identified in 5.4% of children with CM. Hypoglycemia was an independent risk factor for both mortality and neurological sequelae with, compared to those with normal blood glucose, a 2.87-fold greater risk (95% CI: 1.35–6.09) of death and, for survivors, a 3.21-fold greater risk (95% CI: 1.51–6.86) of neurologic sequelae. Interestingly, all hypoglycemic episodes (glucose <2.2 mmol/L) occurred within the first 12 hours of admission, and most (94.7%) glucose levels under the treatment threshold (<3.0 mmol/L) appeared within 24 hours. In those with a blood glucose measurement <3.0 mmol/L in the first 24 hours, low blood glucose levels persisted or recurred as long as 48 hours after admission. Based on their findings, the authors recommend measuring glucose levels every 6 hours during the first 24 hours after admission. If glucose is below 3.0 mmol/L at any time during those hours, monitoring should continue for at least 48 hours. Otherwise, monitoring can stop 24 hours after admission.

These results provide valuable insights into glucose monitoring in children with CM and may improve current WHO guidelines. The study’s strength lies in its large sample size and well-structured assessment of patients, including systematic repeated sampling of each participant.

However, it should be noted that patients were not followed-up after discharge. The authors were thus unable to explore longer-term neurological outcomes.^9^ This limits interpretation of the full impact of hypoglycemia on the incidence of long-term neurological sequelae and post-discharge mortality. Some neurological abnormalities are temporary and may disappear (for example ataxia), others can improve in the short-term (for example cortical blindness and hemiparesis), and severe sequelae (for example spastic tetraparesis) may lead to death in the months after discharge.^8^ On the other hand, more subtle neurocognitive and behavioral deficits that were unnoticed at discharge may only become evident afterwards,^8^ as specific neuropsychological assessment is required for their identification. Irrespective of these and other minor limitations, this study provides very valuable data on the risks associated with single or recurrent hypoglycemia episodes during presentation with CM.

The presented results for mortality and neurological sequelae are consistent with previous research showing a strong association between malaria-related low glucose levels, mortality, and neurological sequalae, either for hypoglycemia (<2.2 mmol/L) or glucose under the treatment threshold (<3.0 mmol/L).^10^ Some studies have compared oral, sublingual, or intravenous glucose administration,^12^^–^^15^ and the overall conclusion was that correction should be done as fast as possible irrespective of the method of administration. Consequently, and considering the low risk of adverse events with glucose administration, we may assume that the benefit of aggressive treatment outweighs potential risks, thus justifying a more flexible threshold (i.e., higher glucose levels) to trigger treatment. Interestingly, there is no direct evidence for preventive glucose administration.

Prompt treatment requires proactive detection. As glucose dynamics are variable, low glucose “treatable” events could be unnoticed without frequent measurements. This means that capillary determinations should be done not only after signs and symptoms appear, but also as part of the routine management of children with CM. In line with the aforementioned study results, frequent and continuous monitoring throughout hospitalization should be done in patients with CM and should continue for 24 hours in those children with consistently normal glucose levels, and for at least 48 hours in those with detected hypoglycemia.

The Chastang et al.^7^ study provides valuable data that could help clinicians in resource-limited settings improve CM management with more efficient use of available resources. Further research may consolidate these findings and eventually influence international recommendations, but there is a need for more clinical studies to better understand the pathophysiology of CM and optimize its management. In addition, developing innovative tools that could complement clinical evaluation and glucose monitoring for improved risk-stratification and prognosis prediction in CM remains a global health priority.

## Financial Disclosure

DT receives the support of a fellowship from “La Caixa” Foundation (ID 100010434). The fellowship code is “LCF/BQ/DR21/11880018”.
